# Waning rate of immunity and duration of protective immunity against diphtheria toxoid as a function of age and number of doses: Systematic review and quantitative data analysis

**DOI:** 10.1080/21645515.2022.2099700

**Published:** 2022-07-21

**Authors:** Noriko Kitamura, Khawater Bahkali, Elvis D Chem, Billy J Quilty, Tansy Edwards, Michiko Toizumi, Lay-Myint Yoshida

**Affiliations:** a Department of Infectious Disease Epidemiology, London School of Hygiene and Tropical Medicine, London, UK; b School of Tropical Medicine and Global Health, Nagasaki University, Nagasaki, Japan; c Department of Pediatric Infectious Diseases, Institute of Tropical Medicine, Nagasaki University, Nagasaki, Japan; d Department of Public Health Intelligence, Public Health Authority, Riyadh, Saudi Arabia; e Graduate School of Biomedical Sciences, Nagasaki University, Nagasaki, Japan

**Keywords:** Antibody, diphtheria, DTP, immunity, systematic review, vaccination

## Abstract

Although the burden of diphtheria has declined greatly since the introduction of vaccines, sporadic outbreaks continue to be reported. WHO recommends booster doses after a primary series, but questions remain about the optimal interval between these doses. We conducted a systematic review and quantitative data analysis to quantify the duration of protective immunity after different numbers of doses. Fifteen cross-sectional seroprevalence studies provided data on geometric mean concentration (GMC). Single-year age-stratified GMCs were analyzed using a mixed-effect linear regression model with a random intercept incorporating the between-country variability. GMC was estimated to decline to 0.1 IU/ml in 2.5 years (95% CI: 0.9–4.0), 10.3 years (95% CI: 7.1–13.6), and 25.1 years (95% CI: 7.6–42.6) after receiving three, four and five doses, respectively. The results drawn from cross-sectional data collected in countries with different epidemiologies, vaccines, and schedules had several limitations. However, these analyses contribute to the discussion of optimal timing between booster doses of diphtheria toxoid-containing vaccine.

## Introduction

Diphtheria is an acute bacterial infectious disease caused by toxigenic *Corynebacterium diphtheriae*. The toxin, secreted by a bacteriophage, induces upper respiratory stenosis or myocarditis. The mortality rate in untreated patients is between 5% and 20%.^1^ The introduction of diphtheria toxoid vaccines reduced disease incidence dramatically in all countries in the world.^2,3^ However, diphtheria is still endemic in low- and middle-income countries. Multiple outbreaks, some of which were large scale, have been reported across the world in the past decade.^4–12^ The incidence of diphtheria has increased more in children older than 5 years of age, than in younger children, which is thought to be due to the increasing three-dose primary series coverage and the lack of booster doses.^13^

Diphtheria toxoid vaccine was introduced into high-income countries between 1930 and 1960^14^ and into low- and middle-income countries after 1974, as part of the Expanded Program of Immunization. It has traditionally been combined with tetanus and pertussis antigens in various formulations of diphtheria–tetanus–pertussis vaccine (DTP) and is now often combined with other antigens, for example, *Haemophilus influenzae B* (Hib), hepatitis B, and inactivated polio vaccine. The current WHO-recommended schedule of vaccination for diphtheria is three primary doses during infancy, a first booster dose between 12 and 23 months, a second booster dose between 4 and 7 years (school-entry), and a third booster dose between 9 and 14 years (school-leaving), though the optimal booster dose timing and interval remain uncertain.^15^ As many low-income countries provide only three primary doses during infancy,^16^ the booster dose schedule is under discussion in light of the increases in diphtheria incidence in some Asian and African countries in the past decade.^15,17–25^

In theory, the optimal timing for booster doses is determined by the waning rate of immunity and hence the duration of protective immunity against the disease after successive doses. Longitudinal data are typically more appropriate than cross-sectional data for evaluating the waning rate of immunity. Several longitudinal studies have followed up individuals’ diphtheria antitoxoid antibody level over years.^26–30^ However, these data were collected among adults above 20 years old. Long-term follow-up studies targeting young children are not available to the best of our knowledge. On the other hand, several cross-sectional seroprevalence data were available, so this study attempted to analyze them.

The objective of this study is to quantify waning rate and duration of protective immunity to diphtheria among children who received a three primary-dose series and each successive booster dose by using published cross-sectional survey data.

## Materials and methods

A systematic review was conducted following PRISMA guidelines to obtain data for analysis on waning immunity (PROSPERO registration number: CRD42020172475). The objective of the systematic review was to extract age-specific data on the prevalence of diphtheria antitoxoid antibodies in populations that received different numbers of DTP vaccine doses.

### Search strategy for identification of studies

The electronic databases MEDLINE, EMBASE, and Global Health were screened from inception to 3 March 2020 using the following text and subject headings: (“corynebacterium” or “diphtheria”) and (“vaccine*” or “vaccination” or “immuni#ation” or “schedule” or “diphtheria toxoid*”) and (“seroepidemiolog*” or “seroepidemiologic studies” or “seroprevalence” or “serology” or “serological survey” or “immune adj3 status”). A manual search was conducted by screening the reference lists of the retrieved full-text articles.

### Inclusion and exclusion criteria

Eligible studies were those that included data on the seroprevalence of diphtheria antitoxoid antibodies among general populations eligible for vaccination following their national immunization program. We also only included studies in which the antibody concentration was measured by the Toxin Neutralization assay (TNT) or adjusted by TNT, as the results obtained by different assays are not directly comparable.^31,32^ No geographical restriction was applied.

Studies were excluded if (i) they were not published in full text, (ii) the full texts were not written in English, (iii) they did not show relevant or adequate information by full-article review, (iv) the same data were used for other eligible studies, (v) data on 1-year age-stratified immunity were not available for at least between aged 1 year and the age at which the first booster dose was scheduled, (vi) seroprevalence was not measured or adjusted by the TNT assay, (vii) the data related to migrants or refugees who had not been included in the vaccination schedule in the study setting, or (viii) the data related to immunocompromised hosts or any specific disease patients.

### Study selection, data extraction, and quality assessment

Two reviewers (NK and KB) screened titles and abstracts of all studies resulting from the search after deduplication managed by Endnote X9 (Clarivate Analytics, US). After the screening, full-text articles were assessed by two reviewers (NK and KB) independently for inclusion or exclusion of each study. Two reviewers (NK and EC) extracted data from the selected studies. Some of the original antibody data were provided by the author of the original articles.

The following information were extracted: study type, publication year, study location (country), study year(s), sample size, sampling method, age (range), number or percentage of seropositive subjects, geometric mean concentration (GMC) of diphtheria antitoxoid antibody, vaccine schedule (recommended age at which vaccine should be given), vaccine coverage by year if available, and year of introduction of primary and booster dose vaccination. A booster dose was defined as any dose after the three primary doses, regardless of the primary-dose series schedule. If numerical data were not available in the full article, an online graphic tool WebPlotDigitizer was used to extract data from published graphic presentations. WebPlotDigitizer was evaluated by several articles and showed excellent consistency between the estimates from the graphics and true values and high levels of inter-coder reliability and validity.^33–35^

Assessment of risk of bias in individual studies was carried out by two investigators (NK and KB) using the tool developed by Hoy et al.^36^ Each study was scored from 0 to 10, with risk determined as low (score >8), moderate (6–8), or high (≤5).

As available vaccination coverage data were limited in the original publications, the national DTP3 annual coverage levels in each country were extracted from the WHO data repository for all countries.^16^ DTP3 coverage was defined as the proportion of those who completed a three primary-dose series of DTP among the population.

### Assumptions about the data

GMC data by single-year age strata were extracted from the original articles and used for the data analysis. We assumed that single-year age stratified GMCs in each country were equivalent to the antibody level measured in the same individual in successive years. We assumed that GMCs increased at the age at which each vaccine dose was scheduled, and GMCs decreased by year similar to the immunity levels in individuals.

Study subjects were assumed to have been offered vaccination according to the vaccination schedule in place in their country at the time of the study. The vaccination status of each study subject was not available. Therefore, the national DTP3 coverage in the birth year of the study subjects was assumed to apply to each birth cohort in each country. Similarly, the booster dose coverage level was not available at individual or national levels, and thus all booster dose coverages were assumed to be the same as DTP3 coverage for each birth cohort. Data on individuals over 20 years of age were excluded as they were not stratified by single-year age.

### Statistical analysis

GMC was calculated by exponentiating the mean log antibody level of subjects and was assumed to decrease exponentially (constantly on a log-scale) to model the waning of immunity.^28,29^

Waning immunity was investigated by analyzing GMC on a logarithmic scale. The time variable was the number of years since the age of the last scheduled vaccination. It was assumed that the waning rates of GMC were different after each successive booster doses, but were the same for all countries, and that the peak immunity levels after receiving a booster dose varied between countries. The peak immunity level might vary due to different vaccine composition or different age at which the vaccine is given. The peak immunity level may also vary by population coverage within a country. A model with variation in peak immunity level allows for more flexibility in the analysis. Therefore, mixed-effect linear regression models with a random intercept incorporating between-country variation were used to model the waning of immunity as a function of age in the cross-sectional data.

The number of doses was included in the model as a categorical variable to adjust the peak immunity level after receiving a booster dose and to assess the modification effect on the waning rate. DTP3 coverage levels were included in the model as a continuous variable, and immunity levels in each birth cohort in each country were adjusted for DTP3 coverage. These two factors were included as a fixed effect as they were assumed to have a constant effect across all countries. The model used for the data analysis was expressed as below.

$$Y_{ij}=\beta_{0}+{\mu_{0}}_{i}+\beta_{1}*\;t_{ij}+\beta_{2}*\;d4_{ij}+\beta_{3}*\;d5_{ij}+\beta_{4}*\;t_{ij}*\;d4_{ij}+\beta_{5}*\;t_{ij}*\;d5_{ij}+\beta_{6}*\;c_{ij}+e_{ij}$$

*where Y*_*ij*_ = log_10_ GMC in country *i* at time *j*, *i* = individual country,

*t* = time since the age of last scheduled vaccine dose (year),

*d* = 3, 4, or 5 doses (categorical variable), *c* = 0% to 100% coverage (continuous variable),

*μ_0i_ =* random effects*, e_ij_ =* error,

Data analyses were conducted using STATA15 (STATA Corp LLC, College Station, TX, USA), and data visualization were conducted using R (R Core Team (2020). Vienna, Austria).

Peak immunity level, waning rate of immunity, and duration of protective immunity after each number of vaccine doses were quantified based on the above prediction model. Waning rate of immunity was assessed by annual percentage decrease of immunity, which was defined as (1 − annual change of immunity) x100%). A fixed effect of the peak log_10_ GMC (intercept of the model) and annual change of log_10_ GMC (slope of the model) were estimated for each number of doses at 90% of DTP3 coverage level with a 95% confidence interval (CI) from the model. As model used log_10_ GMC, back transformation was conducted to obtain the predicted peak immunity and annual change of immunity.

**Figure 1.**
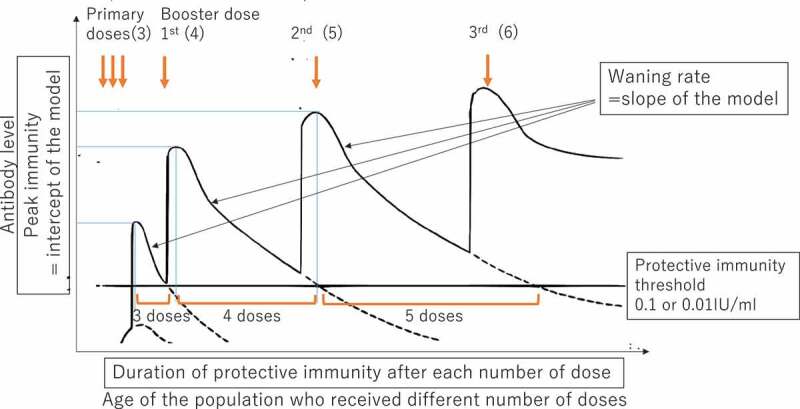
A hypothesized schematic image of the data and analysis and measurement of waning rate of immunity and duration of protective immunity.

Duration of protective immunity was assessed as the period over which immunity (GMC) was estimated to decline to the protective threshold. The times at which GMC was predicted to decline to two standard protective thresholds (0.1 IU/ml and 0.01 IU/ml)^32^ were estimated from the line of best fit assuming that DTP3 coverage was always 90%, which WHO recommends to reach. This predicted time can be considered a measure of duration of protective immunity. Duration of protective immunity is determined by peak immunity level (intercept) and waning rate (slope) after receiving each dose. Figure 1 provides a schematic image showing the hypothesized pattern of peak immunity levels and waning rates after receiving booster doses. The Delta method was used to estimate a 95% CI for the duration of protective immunity.^37,38^

The peak of the immunity curve shows the peak immunity after vaccination. The slope of the graph is waning of immunity after the respective number of doses previously given. Durations of protective immunity are determined by the peak immunity levels after vaccination and waning rate (slope). Figure was adapted from “WHO immunological basis for immunization series: module 3 tetanus”.^51^

## Results

### Systematic review

A total of 1,209 articles were identified on the electronic databases by the search strategy, and 12 articles were identified manually. After removing duplicates, 883 studies were screened. According to the eligibility criteria, 663 articles were excluded, leaving 220 eligible articles. Full articles were examined, and three articles were retained for data analysis.^39–41^

**Figure 2.**
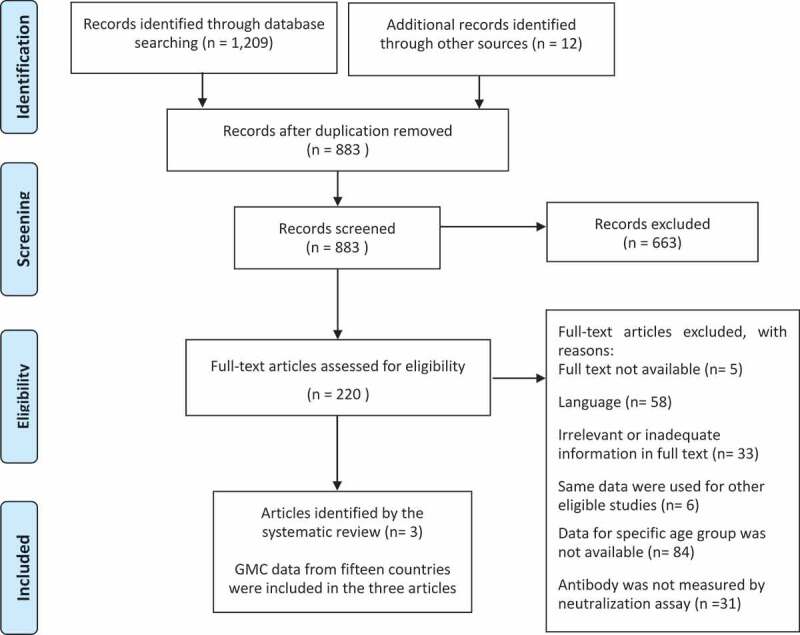
Results of literature search and flow diagram.

GMC data from 15 countries (Czech Republic, Denmark, Finland, France, Hungary, Ireland, Israel, Italy, Latvia, Luxembourg, Norway, Russia, Slovakia, Sweden, and the United Kingdom) were included in three articles. These 15 countries were included in the quantitative analysis of waning rate of immunity (Figure 2).

All countries were in the European region. Data from the 15 above-listed countries were collected as cross-sectional seroprevalence studies between 1995 and 2003. In the Czech Republic, Latvia, Luxembourg, Slovakia, and Sweden, serum samples were collected in population-based surveys. Other countries used residual sera collected during routine laboratory testing. All samples, except for Norway and Russia, were collected as a national serosurvey. Their samples were collected from a wide range of geographical locations within each country, and, to avoid systematic bias, sera likely not to be representative of the population were excluded (e.g. immunocompromised host).^40,41^ In Norway and Russia, the samples were collected at subnational regions.^39^ According to the quality assessment within studies based on Hoy’s criteria, six countries (40%) had a low risk, nine (60%) had a moderate risk, and no study had a high risk of bias (Table 1).

**Table 1.** Summary table of 15 countries included in the quantitative analysis.

Authors	Country	Study year	Age	3 doses	4th dose	5th dose	DTP intro	Booster intro*	Sample size	Sampling method	Hoy’s criteria	DTP3 coverage
			(year)	(month)	(year)	(year)						mean (range)
di Giovine et al.	Czech Rep	2001	0-75+	2,3,4	1.5 (D)	5 (D)	1960	1986	3123	Community	10	100% (96–100%)
Edmunds et al.^31^	Denmark	1995	0-75+	3,5,12	5 (D)		1930s	1996	2989	Residual sera	8	89% (86–91%)
Edmunds et al.^31^	Finland	1995	0-75+	3,4,5	2 (D)	11-13 (d)	1943	1989*	3381	Residual sera	10	95% (90–99%)
Edmunds et al.^31^	France	1995	0-75+	2,3,4	1.5 (D)	5 (D)	1938	NA	2462	Residual sera	8	91% (79–96%)
di Giovine et al.^32^	Hungary	2003	0-60+	3,4,5	3 (D)	6 (D)	1960	1971	2600	Residual sera	8	99% (99–100%)
di Giovine et al.^32^	Ireland	2003	0-60+	2,4,6	4 (D)	12 (d)	1960	1996*	3300	Residual sera	8	66% (36–86%)
di Giovine et al.^32^	Israel	2000	0-60+	2,4,6	1 (D)	7 (D)	1951	1999	3300	Residual sera	10	92% (91–96%)
Edmunds et al.^31^	Italy	1996	0-75+	3,5,11	5-6 (D)	X (d)	1939	NA	3111	Residual sera	8	93% (83–95%)
di Giovine et al.^32^	Latvia	2003	0-60+	3,4.5,6	1.5 (D)	7 (D)	1960	1998*	3300	Community	10	91% (80–98%)
di Giovine et al.^32^	Luxembourg	2001	4-70+	2,4,5	1(D)	5 (D)	1960	1999	3200	Community	8	87% (68–98%)
Skogen et al.^30^	Norway	1994	1-12	3,5,11	11(d)		1942	NA	400	Residual sera (subnational)	6	88% (80–98%)
Skogen et al.^30^	Russia	1994	1-10	3,4.5,6	2 (D)	6 (D)	1958	NA	264	Residual sera (subnational)	6	76% (73–79%)
di Giovine et al.^32^	Slovakia	2003	0-60+	2,4,10	2 (D)	5 (D)	1960	1998	3300	Community	10	99% (99–100%)
Edmunds et al.^31^	Sweden	1995	0-75+	3,5,11	10 (D)		1951	NA	3633	Community	10	99% (99–99%)
Edmunds et al.^31^	UK	1996	0-75+	2,3,4	3.3 (D)	15 (d)	1940	1994*	3224	Residual sera	8	69% (41–94%)

X: Decennial booster dose.

D and d refer to high and low dose of diphtheria toxoid, respectively.

†Final sample size was not reported for some studies (Hungary, Ireland, Israel, Latvia, Luxembourg and Slovakia). For those studies, the target sampling size was used for sample size.

*Multiple booster doses were used in the majority of countries. The introduction years of the booster dose were for the fifth dose.

§Hoy’s criterion score were used to assess the quality of the prevalence study for the systematic review.

Data from the 15 countries, including the target population, sample size, vaccination schedule, and introduction year of the booster doses, are summarized in Table 1. Age at first booster dose varied from 12 months to 10 years, and the total number of doses varied from four to seven. As the number of countries providing more than five doses of DTP was limited, we quantified waning rate of immunity and duration of protective immunity after three, four, and five doses only. The available data for analysis, such as age range and sample sizes in each country, are summarized in Table 2.

**Table 2.** Age ranges and original sample sizes for the 15 countries population included in the quantitative analysis for waning rate of immunity. ^30–32^

Country		After 3 doses	After 4 doses	After 5 doses	After 6 doses
Czech Republic	Age range (year)	1	2-4	7-19	ns
	Sample size	56	283	1289	
Denmark	Age range (year)	2-4	6-16	ns	ns
	Sample size	287	783		
Finland	Age range (year)	1	2-10	13-17	ns
	Sample size	100	854	470	
France	Age range (year)	1	2-5	7-10	12-15
	Sample size	45	200	173	173
Hungary	Age range (year)	ns	3-5	6-10	13-14
	Sample size		300	500	200
Ireland	Age range (year)	ns	4-9	13-19	ns
	Sample size		600	700	
Israel	Age range (year)	ns	1-6	ns	ns
	Sample size		600		
Italy	Age range (year)	1-4	8-15	ns	ns
	Sample size	359	695		
Latvia	Age range (year)	ns	2-6	8-12	14-19
	Sample size		500	500	500
Luxembourg	Age range (year)	ns	4	ns	9-11
	Sample size		100		300
Norway	Age range (year)	1-10	12	ns	ns
	Sample size	336	29		
Russia	Age range (year)	1	3-5	6-8	9-10
	Sample size	15	99	79	58
Slovakia	Age range (year)	1	3-4	6-10	ns
	Sample size	100	200	500	
Sweden	Age range (year)	1-9	10-19	ns	ns
	Sample size	528	724		
UK	Age range (year)	ns	6-13	15-17	ns
	Sample size		763	299	

ns: no samples were included in the analysis for respective countries and doses.

The sample size in this table for Ireland, Israel, Hungary, Latvia, Luxembourg, and Slovakia were the number of samples that were planned to be collected as final sample sizes were not available in the cited publications.

### Waning of immunity and duration of protective immunity

**Figure 3.**
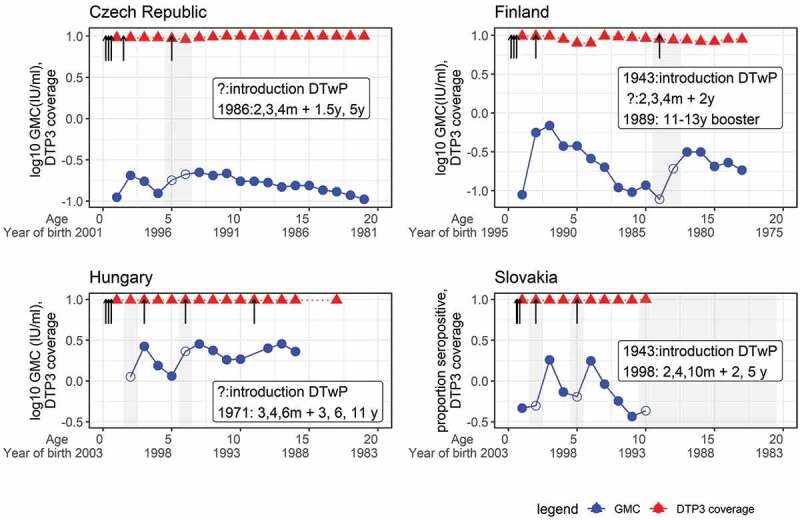
Seroprevalence and DTP3 coverage by age and year of birth in data included in the analyses.

Before conducting the analysis, all the GMC data were plotted by birth cohorts in each country. Some birth cohorts were excluded because they were over the target age of the booster doses when those doses were introduced. The antibody level was expected to reach a peak within a year of each scheduled dose. However, some delayed peaks were observed 1 to 2 years after the scheduled booster dose age. As delayed peaks affect the waning rate, data before the peak were removed. Figure 3 shows four countries’ original data and how the data were treated before analysis in the regression model.

These figures are a descriptive aid to show which data were used for the regression analysis. Data from four countries are shown here. Some birth cohorts shaded in gray were removed for various reasons: (1) some birth cohorts were too old to receive booster doses when they were introduced, e.g., ≥10 years old birth cohort in Slovakia, and (2) GMC was lower than the peak at the age of the scheduled booster dose, e.g., the birth cohort aged 5-6 years in the Czech Republic and the birth cohorts aged 11 and 12 years in Finland. The birth cohorts removed from the analysis were expressed as hollow circles and the remaining cohorts included in the analysis were shown as solid circles. Solid circles indicate GMC, and triangles indicate national DTP3 coverage. Arrows indicate the vaccination schedule in each country.

**Figure 4.**
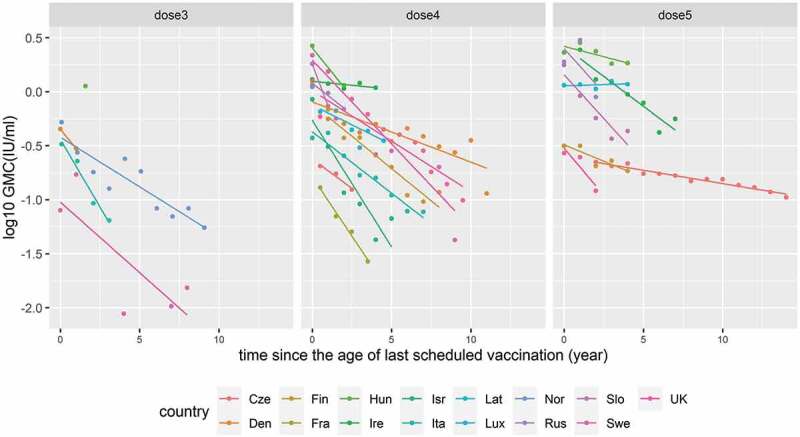
Declining trend of GMC over time after different numbers of DTP doses were given in 15 countries: Czech Republic, Denmark, Finland, France, Hungary, Ireland, Israel, Italy, Latvia, Luxembourg, Norway, Russia, Slovakia, Sweden, and the United Kingdom.

GMCs in the 15 countries were plotted by year since the age of last scheduled vaccination separately by number of doses. There was some heterogeneity in waning rate by country expressed as a slope of simple linear regression (Figure 4).

Each observed value of GMC was plotted over time for each country on a log_10_ scale. Predicted lines were drawn by the simple linear regression of immunity over time but not adjusted for coverage.

The average peak GMC level in 15 countries was expressed as an intercept predicted by the mixed-effect linear regression model. The average waning rate of GMC was expressed as a slope predicted by the model. GMC declined significantly by year after the last scheduled primary and booster dose (*p-value <0.01*). The peak GMC levels were 0.21 IU/ml, 0.71 IU/ml, and 0.58 IU/ml; the annual percentage decrease of GMC was 26%, 17%, and 7% per year, respectively, after three, four, and five doses. GMC was predicted to decline to 0.1 IU/ml in 2.5 years, 10.3 years, and 25.1 years and predicted to decline to 0.01 IU/ml in 10.0 years, 22.5 years, and 58.0 years, respectively, after receiving three, four and five doses (Table 3).

**Table 3.** The peak GMC level, annual percentage decrease of GMC level, and duration of protective immunity after three, four, and five doses: Duration of protective immunity was estimated as the time at which GMC declined to 0.1 IU/ml or 0.01 IU/ml.

	Peak GMC level (intercept)		Annual percent decrease (slope)		Duration of protective immunity (intercept-threshold/slope)			
	IU/ml	(95% CI)	%	(95% CI)	Declined to 0.1 IU/ml		Declined to 0.01 IU/ml	
					year	(95% CI)	year	(95% CI)
After 3 doses	0.21	(0.13, 0.35)	26%	(20.5, 31.9%)	2.5	(0.9, 4.0)	10.0	(7.4, 12.5)
After 4 doses	0.71	(0.45, 1.12)	17%	(12.2, 21.9%)	10.3	(7.1, 13.6)	22.5	(16.1, 29.0)
After 5 doses	0.58	(0.36, 0.94)	7%	(1.7, 11.5%)	25.1	(7.6, 42.6)	58.0	(16.6, 99.4)

*Peak GMC level (intercept) and annual percentage decrease (slope) after each vaccine dose were estimated assuming at 90% of DTP3 coverage with 95% CI from the model. The mean peak GMC level (IU/ml) was calculated by 10exp(“intercept”). Annual percentage decrease was calculated by (1 − 10exp(“slope”)) x 100%).

## Discussion

In this study, we quantified the peak immunity and waning rate of anti-diphtheria antitoxoid antibodies after different numbers of DTP doses. We also estimated duration of protective immunity. The prediction was conducted using 15 countries’ data in Europe. The estimated duration of protective immunity may be considered an optimal booster dose interval and will be useful for countries where additional booster doses need to be introduced. Our study found that GMC was estimated to decline to 0.1 IU/ml in 2.5 years after three-dose primary series. This indicates that the currently recommended first booster dose at 12–23 months of age is reasonable.^42^ In addition, GMC remained above 0.1 IU/ml in 10.3 years after four doses. This result justifies DTP schedules in some countries, such as Finland, the United Kingdom, and Japan, which provides the fifth dose about 10 years after the fourth dose. In this study, GMC remained above 0.1 IU/ml for 25.1 years after five doses, which were completed between age five and fifteen. Although our study did not measure the duration of protective immunity after the sixth dose, 25.1 years of protection is similar to a previously estimated duration of protection after six doses of DTP. A cross-sectional seroprevalence study in the Netherlands, which provides a sixth dose of DTP at 9 years of age, estimated that individuals would be protected until they were 37 years old.^43^ While WHO anticipated more than six doses would not be required in many populations,^44^ it is still unclear whether additional doses are required for the middle-aged population.

We previously measured the duration of protective immunity using 2-year cohort data in a well-vaccinated community in Vietnam.^45^ This study showed that IgG remains above 0.1 IU/ml for 4.3 years (95%CI:3.5,5.3) after the fourth dose of DTP was given at 18 months of age. This result supports the recommendation for a school-entry booster dose. A cross-sectional seroprevalence study in South India showed that the proportion of children whose IgG levels were above 0.1 IU/ml declined from 47.4% at age five to 12.6% at age 17 after fifth dose of vaccine was given at age five.^46^ Indian data showed much faster waning rate than estimated in this review. The reason for this difference might be low vaccination coverage in older age groups in the Indian population. Truelove et al. conducted a systematic review and pooled analysis to estimate waning rate of immunity by using a mixed-effect log-linear regression model.^47^ This study analyzed cross-sectional seroprevalence data with 888 age group observations from 62 studies. The original studies were conducted in Europe, Asia, and North and South America between 1962 and 2016. Their estimated annual decline of proportion of immune (above 0.1IU/ml) by age since vaccination of DTP was 0.75% per year of age (95% CI, 0.25–1.24%). We have estimated the annual decline of proportion of immune (data not shown) along with waning rate of GMC levels, but our results indicated much more rapid decline than their estimate. Potential reasons for the difference include that their analysis combined serological data measured by different assays (i.e. ELISA and TNT), different age groups, different vaccine doses previously given, different study periods, and different geographical areas. Discrepancies between the results obtained from Asia and Europe cannot be explained by single factors and are probably attributable to multiple epidemiological differences between regions or variation of source data and estimation methods.

There are several limitations to this analysis. There are differences between countries in terms of vaccine composition and schedule, vaccination coverage, (Table 1) background diphtheria incidence, and the original type of serological assays, while we assumed waning rates were the same in all the countries analyzed. Ideally, these additional factors affecting immunity should be adjusted but it was not possible to quantify them, except for the vaccination coverage. The vaccination status of the study populations was not available and might have been different from the national coverage. Further, data on booster dose coverage were also not available, which may have a significant influence on immunity in later life. It was assumed that the booster dose coverage was the same as DTP3 in each birth cohort, although this coverage is likely to be lower than DTP3. This assumption may have led to an underestimation of the duration of protective immunity if all doses were actually received. DTP3 coverage did not modify the GMC level in our analysis; however, this might be because unadjusted factors, mentioned above, masked the effect of coverage. The average peak immunity after the fifth dose were not increased from the fourth dose. This might have occurred because of low fifth dose coverage, delayed timing of vaccination, or low immune response after long interval from the fourth dose. The wide 95% CI of the predicted waning rate and that of the predicted duration of protective immunity, especially after fifth dose, are attributable to the heterogeneity by country and the limited sample size. Therefore, we cannot make firm conclusions from the current results.

As an additional limitation, it has been suggested that cutaneous diphtheria may play a role in maintaining protective immunity to diphtheria, particularly in tropical countries.^48^ Since 1997, the Hib vaccine has been combined with DTP and used worldwide.^49^ Modified diphtheria toxoid is used as a protein carrier in conjugate Hib vaccine, which has been shown to increase the diphtheria antitoxoid antibody level among recipients.^50^ Therefore, the results derived from European data collected between 1995 and 2003, with relatively high infant vaccination coverage and homogeneous populations in temperate climate, might not be generalizable to the current populations in tropical settings. The study also has several strengths. The currently available data were searched by systematic review. National seroprevalence surveys with a large sample size were used for this analysis. The risk of bias of the source data was confirmed as low or moderate according to the Hoy’s assessment criteria. Except for Norway and Russia, sampling methods were quite similar in all countries as original surveys were conducted as a multi-country study in Euro-Surveillance Network. We estimated the waning rate of vaccine-derived immunity by the number of vaccine doses, which does not appear to have been reported before. The method using already available cross-sectional serology data is simpler and cheaper than carrying out a longitudinal study to provide additional information for the vaccination schedule, notwithstanding the several limitations mentioned above.

## Conclusions

We estimated the waning rate of immunity and duration of protective immunity after consecutive doses of DTP from cross-sectional seroepidemiological data with the assumption that the study participants were vaccinated according to the reported vaccination coverage. Our results indicate potential optimal booster dose intervals for diphtheria toxoid-contained vaccine. However, the several assumptions made in the method increased the risk of inaccuracy; therefore, the conclusions drawn here need to be treated cautiously. The results should be taken into consideration along with the various factors that determine appropriate vaccination schedules, including waning of other co-administered vaccine components, especially pertussis, and the epidemiological background in each country.
